# The Built Environment Assessment of Residential Areas in Wuhan during the Coronavirus Disease (COVID-19) Outbreak

**DOI:** 10.3390/ijerph19137814

**Published:** 2022-06-25

**Authors:** Heli Lu, Menglin Xia, Ziyuan Qin, Siqi Lu, Ruimin Guan, Yuna Yang, Changhong Miao, Taizheng Chen

**Affiliations:** 1 College of Geography and Environmental Science/Key Research Institute of Yellow River Civilization and Sustainable Development & Collaborative Innovation Center on Yellow River Civilization of Henan Province, Henan University, Kaifeng 475004, China; luheli@vip.henu.edu.cn (H.L.); xmllin@henu.edu.cn (M.X.); qzyuan@henu.edu.cn (Z.Q.); 490707753@henu.edu.cn (R.G.); yangynn@henu.edu.cn (Y.Y.); chhmiao@henu.edu.cn (C.M.); 10130043@vip.henu.edu.cn (T.C.); 2 Key Laboratory of Geospatial Technology for the Middle and Lower Yellow River Regions (Henan University), Ministry of Education/National Demonstration Center for Environment and Planning, Henan University, Kaifeng 475004, China; 3 Henan Key Laboratory of Earth System Observation and Modeling, Henan University, Kaifeng 475004, China; 4 Henan Dabieshan National Field Observation and Research Station of Forest Ecosystem, Henan University, Kaifeng 475004, China; 5 Department of Geography, University of Connecticut, Storrs, CT 06269-4148, USA

**Keywords:** Wuhan, COVID-19 outbreak, environmental factors, residential area layout, natural ventilation potential

## Abstract

The COVID-19 epidemic has emerged as one of the biggest challenges, and the world is focused on preventing and controlling COVID-19. Although there is still insufficient understanding of how environmental conditions may impact the COVID-19 pandemic, airborne transmission is regarded as an important environmental factor that influences the spread of COVID-19. The natural ventilation potential (NVP) is critical for airborne infection control in the micro-built environment, where infectious and susceptible people share air spaces. Taking Wuhan as the research area, we evaluated the NVP in residential areas to combat COVID-19 during the outbreak. We determined four fundamental residential area layouts (point layout, parallel layout, center-around layout, and mixed layout) based on the semantic similarity model for point of interest (POI) picking. Our analyses indicated that the center-around and point layout had a higher NVP, while the mixed and parallel layouts had a lower NVP in winter and spring. Further analysis showed that the proportion of the worst NVP has been rising, while the proportion of the poor NVP remains very high in Wuhan. This study suggested the need to efficiently improve the residential area layout in Wuhan for better urban ventilation to combat COVID-19 without losing other benefits.

## 1. Introduction

Since the first case of infection was discovered in December 2019, coronavirus disease 2019 (COVID-19) has spread worldwide. There are over 255.1 million confirmed cases worldwide and over 5.12 million deaths, involving more than 200 countries and regions [1]. Variant strains have been found in many countries and regions [2,3], such as India, the United States, France, and Germany, and more studies have shown that variant strains are associated with higher mortality [4]. Many countries have begun encouraging vaccinations, promoting education, and strengthening food cold-chain testing [5,6,7,8]. They can be classified into two major categories based on their prevention and control logic and essential characteristics [9,10,11,12]. One is the containment strategy, adopted by countries such as China and North Korea, and the other is the mitigation strategy, adopted by the United States, Germany, Vietnam, and the United Kingdom. To date, fighting the pandemic is still a top issue that faces the world [13,14].

An increasing number of studies have shown that environmental factors, such as temperature, humidity, light, air particles, and airborne microorganisms strongly relate to the spread of COVID-19 [15,16,17,18,19,20,21,22,23,24]. Ijaz et al. observed that a moderately humid environment was conducive to the survival of the virus. Furthermore, the half-life of the coronavirus in a low-temperature environment of 6 °C ± 1 °C is significantly higher than that at normal room temperature, indicating that low temperatures under the same humidity are conducive to the survival of the virus [25]. Holtmann et al. also concluded through a statistical analysis of data at the early stage of development of the Barcelona area, that low temperatures are beneficial to the spread of COVID-19 [26]. Ward et al. observed that the decrease in relative humidity in Sydney led to an increase in the number of COVID-19 infections [27]. In addition to the temperature and humidity, Schuit et al. concluded that light could promote the attenuation of the activity of COVID-19 [28]. A study by Pyankov et al. concluded that Middle East Respiratory Syndrome (MERS) virus activity decays faster under typical environmental conditions in the Middle East (38 °C, relative humidity (RH) = 24%) than in an indoor environment (25 °C, RH = 79%), and pointed out that environmental factors can affect its survival [29]. This impact may be one of the reasons why the spread of MERS is much lower than that of severe acute respiratory syndrome and COVID-19. It is also believed that airborne particulate matter (PM) or airborne microorganisms are related to infectious diseases [30,31], and COVID-19 is a pandemic. In addition, the spread of COVID-19 is related to environmental factors [32,33]. In a separate study, the coronavirus remained infectious in water and sewage for several days to several weeks [34,35,36,37]. The potential spread of COVID-19 increases with poor personal hygiene [38].

Aerosol transmission refers to a droplet nucleus composed of proteins and pathogens that lose water during the air suspension process, forming a droplet nucleus, which can float to a distance in the form of aerosols, causing long-distance transmission [39,40]. Aerosols can remain in the air for several hours through tiny particles [41,42,43], and related studies have shown that aerosols can spread up to 13 km [44]. In addition, the scope of aerosol transmission is also significantly different from direct transmission, not only in the indoor space of the building, but also in the outdoor urban space [45,46,47]. In July 2020, 239 scientists pointed out in an open letter to the WHO that the COVID-19 aerosol can survive in the air for several minutes to several hours [48]. By controlling the aerosol transmission of COVID-19, urban ventilation can be promoted by rationally designing and optimizing the urban layout, because good urban ventilation can quickly reduce the aerosol concentration in an area. Furthermore, it can improve the natural ventilation of the building, thereby indirectly preventing the space from contributing to the direct spread of the virus. When the ventilation of residential areas is adequate, the probability of people being infected by an aerosolized virus can be reduced by increasing the natural ventilation to reduce the concentration of the COVID-19 virus per unit area [49]. In the “Recommendations on Residential Building Measures for Home Prevention and Control in Response to the New Coronary Pneumonia Epidemic”, three of the fifteen prevention and control measures included the importance of natural ventilation and ensuring air quality [50], confirming the necessity of the wind environment in residential areas. 

Buildings in a residential area and the surrounding physical environment together form the micro-environment of the area. The ventilation environment is an important component of the micro-environment of residential areas and has a significant impact on outdoor pedestrian activities and physical health [51,52]. Erell et al. proposed a set of methods for the climate-adaptive design of urban canyons and indicated that controlling climate factors, such as the wind through an urban space form, landscape structures, and other factors, could improve the comfort of the outdoor environment [53]. Hamnett’s quantitative analysis of the wind environment in urban residential areas found that the wind speed ratio of different building layouts could reach 0.17 at most [54]. Kubota et al. took 22 urban residential areas with different building densities and plot ratios as the research objects to compare the differences in the wind environment [55]. Lin et al. studied the outdoor wind environment based on the layouts of center-around buildings and courtyard-style communities [56]. Peng et al. simulated the wind environment and found that reducing the building height and increasing the number of buildings could be conducive to creating a good building wind environment while keeping the total building area unchanged [57]. However, the micro-environment of residential areas is not only affected by local weather and meteorological conditions, such as solar radiation, wind speed, and other natural factors, but is also affected by factors, such as the greening rate, building layout, and building coverage in residential planning [58]. Moreover, the layout of different types of buildings has a significant impact on the micro-environment of residential areas [59,60]. There are significant differences in the ventilation conditions of residential areas between the different spatial layouts [61,62].

The POI (Point of Interest) generally refers to the point data in electronic internet maps and basically includes four attributes: name, address, coordinates, and category [63,64]. It can be abstracted into points for management, analysis, and calculation. With the popularization of the internet electronic map service, POI forms the basis of content for a growing number of mobile and social media applications. There are many ways to pick POI data, among which the semantic similarity model is an important method. The semantic similarity model generally refers to calculating the similarity between ontology concepts. Compared with the traditional retrieval methods, the semantic similarity model takes concepts as the basic unit of information expression and can more accurately understand the semantics of the words expressed in retrieval requests [65]. Therefore, the semantic similarity model for POI picking has better stability and accuracy than the traditional crawler algorithm. In this paper, we aim to analyze the relationship between the spatial layouts and natural ventilation potential (NVP) to assess the role of residential areas in resisting the spread of the COVID-19 based on POI data. This study contributes to the literature by highlighting the environmental factors of ventilation for combating the COVID-19.

## 2. Materials and Methods

### 2.1. COVID-19 Outbreak in Wuhan

**Figure 1:**
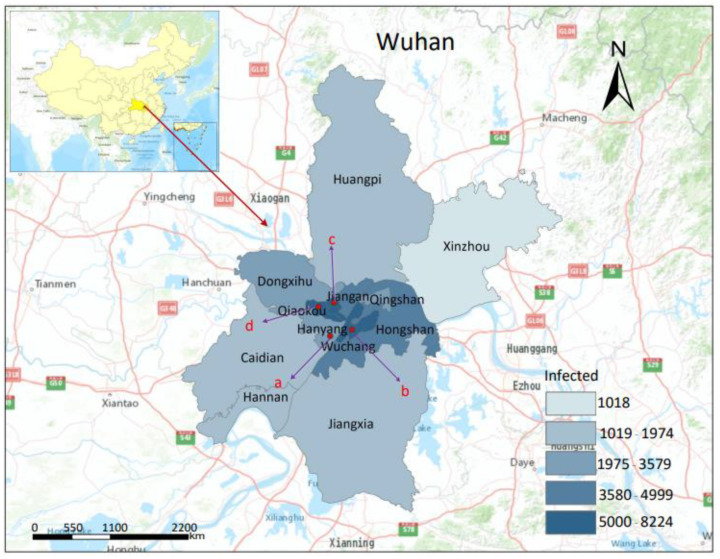
Map showing the location of Wuhan, 13 districts, 4 representative layouts of residential area (a: points layout, b: parallel layout, c: center-around layout, d: mixed layout), and infection numbers during the COVID-19 outbreak.

At the end of 2019, COVID-19 broke out in Wuhan and quickly spread throughout China, causing heavy losses to the country’s stable social and economic development and the safety of people’s livelihoods [66]. On 31 December 2019, when the Wuhan Health Commission announced the “Emergency Notice on Reporting the Treatment of Unexplained Pneumonia”, the cumulative number of confirmed cases was 27; however, the number of patients with symptoms was more than 200. The potential causes of viral exposure may have exceeded 600 people. On 23 January 2020, when Wuhan was locked down, the Health Commission reported a total of 814 confirmed cases; however, the number of patients with symptoms was ~17,000. The number of people who had been exposed to the virus may have been as high as 30,000, and many potential cases were imported into all parts of the country through the Spring Festival [67,68,69]. On 29 February 2020, a total of 49,122 confirmed cases were reported in Wuhan. Among them, 4300 cases were in Jiang’an District, 7290 cases were in Jianghan District, 7239 cases were in Qiaokou District, 3579 cases were in Hanyang District, 8224 cases were in Wuchang District, 2958 cases were in Qingshan District, 4999 cases were in Hongshan District, 2588 cases were in Dongxihu District, 1974 cases were in Caidian District, 1661 cases were in Jiangxia District, 1775 cases were in Huangpi District, 1018 cases were in Xinzhou District, and 1517 cases were in the Wuhan Development Zone (Hannan) (as of 29 February 2020) [70]. The areas with the higher risk levels were mainly distributed in the Wuchang, Jianghan, and Qiaokou districts, while the areas with lower risk levels were mainly distributed in the Caidian, Jiangxia, Huangpi, and Xinzhou districts (Figure 1).

### 2.2. Data

#### 2.2.1. Wind Data

This study mainly used the environmental conditions when Wuhan was locked down to analyze residential areas’ natural ventilation potential (NVP). The wind direction data used were obtained from the National Meteorological Science Data Center (http://data.cma.cn/site/index.html (accessed on 20 October 2021)), and the initial daily wind direction data on the COVID-19 outbreak from December 2019 to March 2020 was selected for the integrated statistics. According to statistics, the number of days when the wind direction is northerly is the highest, the frequency reaches 47.30%, and the average wind level is also the highest, at 3.60; the northeast wind is second, with a frequency of 20.27% and an average wind level of 3.13, followed by an easterly wind and southeasterly wind, where the frequencies are 18.92% and 13.51%, respectively, and the wind levels are 2.57 and 2.40, respectively. Therefore, this study selected the northerly, northeasterly, easterly, and southeasterly winds, which are the top four in terms of their frequency and wind levels, as the key wind directions of this study.

#### 2.2.2. POI Data

Recently, with the rapid development of IT and communication technologies, such as the internet, cloud computing, and triple play, big network data have shown to have an increasingly huge influence and role, and the point of interest (POI) data, as an important network data resource, are increasingly valued by the people and widely used [71,72]. In this research, an ontology-based conceptual-semantic-similarity calculation model was used to capture the POI data [73,74,75] as follows:

The semantic distance, *Dis* ($C_{1}$, $C_{2}$), between the concepts $C_{1}$ and $C_{2}$, is expressed as the number of edges where the shortest path of these two concepts is located on the ontology tree. The formula for the semantic-distance influence factor, $IF_{Dis}$, is as follows:

(1) $$IF_{Dis}=\frac{\tau }{\tau +Dis^{2}\left(C_{1,}C_{2}\right)}$$

where τ is the adjustment factor and is a real number greater than 0; the larger the semantic distance between two concepts (the shorter the shortest path between the concepts), the smaller the semantic-distance influence factor $IF_{Dis}$, the more similar the concepts are when small.

The concept density between the concepts $C_{1}$ and $C_{2}$ is expressed as the number of direct child nodes contained in the nearest common ancestor of the two concepts in the ontology tree. The formula for the concept-density influence factor, $IF_{Den}$, is as follows:

(2) $$IF_{Den}=\frac{Density\left(C_{s}\right)}{Density\left(O\right)}$$

where $C_{s}$ is the closest common-ancestor concept node of the concepts $C_{1}$ and $C_{2}$; $Density(C_{s}$) represents the number of direct-child concept nodes of concept $C_{s}$; $Density\left(O\right)$ is the largest number of sub-concept nodes in the entire ontology tree. The more direct the child nodes contained in the nearest common-ancestor concept node, the greater the concept-density impact factor and the greater the semantic similarity.

The concept’s depth of concept C is expressed by the number of edges on the shortest path between the concept node and the root node of the ontology tree. The formula for the concept-depth impact factor $IF_{Dep}$ is as follows:

(3) $$IF_{Dep}=\frac{1}{2}\left(\frac{ConDep\left(C_{1}\right)+ConDep\left(C_{2}\right)}{\left|ConDep\left(C_{1}\right)+ConDep\left(C_{2}\right)\right|+2\times ConDep\left(O\right)}+\frac{ConDep\left(C_{s}\right)}{ConDep\left(O\right)}\right)$$

where $ConDep\left(C\right)$ represents the level depth of concept tree C in the ontology tree, that is, the length of the shortest path from concept C to the root node of the ontology tree. $ConDep\left(O\right)$ represents the maximum level of depth of all the concepts in the entire ontology tree, and $ConDep\left(C_{s}\right)$ represents the level depth of the nearest common-ancestor concept node $C_{s}$ in the concept trees $C_{1}$ and $C_{2}$. In the ontology tree, the deeper the concept, the more specific it is. Therefore, in the case of the same semantic distance, the similarity of the two concept nodes that are far from the root node of the ontology tree is greater than the similarity of the two concept nodes close to the root node.

The concept-coincidence degree between concepts $C_{1}$ and $C_{2}$ is expressed as the number of common ancestor nodes in the ontology tree contained between the concepts. The formula for the concept-coincidence factor ${\mathrm{IF}}_{\mathrm{coi}}$ is as follows:

(4) $${\mathrm{IF}}_{\mathrm{coi}}=\frac{\mathrm{count}\left(\mathrm{Up}\left(C_{1}\right)\cap \left(\mathrm{Up}\left(C_{2}\right)\right)\right)}{\max\left(ConDep\left(C_{1}\right),ConDep\left(C_{2}\right)\right)}$$

where $\mathrm{count}\left(\mathrm{Up}\left(C_{1}\right)\cap \left(\mathrm{Up}\left(C_{2}\right)\right)\right)$ represents the number of common ancestor nodes contained in the concept nodes $C_{1}$ and $C_{2}$, and $\max\left(ConDep\left(C_{1}\right),ConDep\left(C_{2}\right)\right)$ represents the maximum value of the level depth in the concept nodes $C_{1}$ and $C_{2}$. If the number of common ancestor nodes of two concept nodes is greater, the degree of overlap of their concepts is higher, and the similarity is greater.

Five semantic relationships were considered in the research: Synonym, Induced-By, Inheritance (Is-a), Part-of, and Coordinate-term-of. The formula of the semantic-relationship influence factor ${\;\mathrm{IF}}_{\mathrm{Rel}}$ is as follows:

(5) $${\mathrm{IF}}_{\mathrm{Rel}}=\frac{\sum_{i=1}^{N_{S}}Value\left(C_{i},\;F_{i}\right)}{N_{S}}$$

$$Value\left(C_{i},\;F_{i}\right)=\left\{\begin{array}{c} 1\;\;\;\;\;\;\;\;\;\;\;\;\;Synonym\left(C_{i},\;F_{i}\right)\\ \frac{1}{2}\;\;\;\;Induced-By\left(C_{i},\;F_{i}\right)\;\;\\ \frac{1}{3}\;\;\;\;\;\;\;\;\;\;\;\;\;\;\;\;\;\;\;Is-a\left(C_{i},\;F_{i}\right)\\ \frac{1}{3}\;\;\;\;\;\;\;\;\;\;\;Part-of\left(C_{i},\;F_{i}\right)\\ \frac{1}{4}\;\;\;\;Coordinate-term-of\left(C_{i},\;F_{i}\right) \end{array}\right.$$

where $N_{S}$ represents the number of the shortest paths between concepts $C_{1}$ and $C_{2},\;C_{i}$ represents the *i*-th concept node on the shortest path, and $F_{i}$ represents the parent concept of $C_{i}$. $\mathrm{Value}\;\left(C_{i},\;F_{i}\right)$ represents the weight of the directed edge between concept node $C_{i}$ and its parent concept node $F_{i}$. Combining the above five influencing factors, the formula for calculating the concept of semantic similarity between concepts $C_{1}$ and $C_{2}$ $\mathrm{sim}\left(C_{1},C_{2}\right)$ is as follows:

(6) $$\mathrm{sim}\left(C_{1},C_{2}\right)=f_{1}\times {\mathrm{IF}}_{\mathrm{Dis}}+f_{2}\times {\mathrm{IF}}_{\mathrm{Den}}+f_{3}\times {\mathrm{IF}}_{\mathrm{Dep}}+f_{4}\times {\mathrm{IF}}_{\mathrm{coi}}+f_{5}\times {\mathrm{IF}}_{\mathrm{Rel}}$$

where $f_{1}$, $f_{2}$, $f_{3}$, $f_{4}$, and $f_{5}$ are the adjustment factors. In order to obtain the topic semantic-weight vector, topic concept C was first determined, and then the concept of semantic similarity between each topic word in topic vector TK and topic concept C was calculated according to Formula (6), and the topic semantic-weight vector is $\mathrm{WTK}\;\left\{{\mathrm{wtk}}_{1},{\;\mathrm{wtk}}_{2},\;\ldots {\mathrm{wtk}}_{i}\ldots ,{\;\mathrm{wtk}}_{n}\right\}$. The WTK calculation formula is as follows:

(7) $$\mathrm{WTK}=\left(\mathrm{sim}\left(C,{\mathrm{tk}}_{1}\right),\mathrm{sim}\left(C,{\mathrm{tk}}_{2}\right),\ldots \ldots ,\mathrm{sim}\left(C,{\mathrm{tk}}_{n}\right)\right)$$

where ${\mathrm{wtk}}_{i}$ represents the topic semantic-weight of the *i*-th topic word in the topic vector, that is, the semantic similarity value between topic concept C and topic word tk.

The POI data on the AutoNavi map, using the Mars coordinate system, were used in the research. To facilitate the subsequent data analysis, a conversion algorithm was used to convert the Martian coordinate system to the WGS84 coordinate system during the data visualization. Then, the satellite image layer of the Gaode Map [76] was used to locate the 9368 residential areas in Wuhan, manually identify and confirm the spatial layout of the residential areas, and finally aggregate the 8330 residential areas used in the study.

### 2.3. NVP for Residential Area Layout

#### 2.3.1. Wind Resistance Coefficient (WRC)

The wind resistance coefficient is predominantly affected by the height of a building. The horizontal wind speed of the residential area is derived from the following formula, which provides the wind resistance coefficient [77,78]:

(8) $$V=V_{0}{\left(\frac{Z}{Z_{0}}\right)}^{0.25\;}$$

where V and $V_{0}$ are the wind speeds at heights Z and $Z_{0}$, respectively, and $Z_{0}$ is the reference height. The amount of ventilation from the ground elevation,$\;Z_{b}$ (referring to the elevation of the ground or roof), to the control height, $Z_{e}$, is:

(9) $$Q_{b}=\int_{Z_{b}}^{Z_{e}}V_{0}{\left(\frac{Z}{Z_{0}}\right)}^{0.25}dz=\frac{V_{0}}{1.25}{\left(\frac{Z}{Z_{0}}\right)}^{1.25}\left|{\;}_{Z_{b}}^{Z_{e}}\right.$$

(10) $$\;\;Q_{0}=\frac{V_{0}}{1.25}\left[{\left(\frac{Z_{e}}{Z_{0}}\right)}^{1.25}-{\left(\frac{Z_{b}}{Z_{0}}\right)}^{1.25}\right]$$

The unconstrained (free) ventilation rate is

(11) $$Q_{0}=\frac{V_{0}}{1.25}{\left(\frac{Z_{e}}{Z_{0}}\right)}^{1.25}$$

Then, the ratio of the influence of a building with height, $Z_{e}$ on ventilation is

(12) $$\varepsilon_{b}=\frac{Q_{0}}{Q_{b}}$$

The wind resistance coefficient, $K_{b}$, can be obtained according to the reduction ratio of ventilation as follows:

(13) $$K_{b}=\varepsilon_{b}=\frac{Z_{e}{}^{1.25}}{Z_{e}{}^{1.25}-Z_{b}{}^{1.25}}$$

When $Z_{b}$ = 0, $K_{b}$ = 1, and when $Z_{b}$ ≥ $Z_{e}$, $K_{b}$ = 100,000, Equation (13) can be used to determine the wind resistance coefficients of residential areas.

#### 2.3.2. Spatial Layouts of Residential Areas and NVP

Firstly, the layout of the residential area was determined based on a comprehensive consideration of factors, which is described by extracting the topological graph of the spatial relationships between the residential buildings [79,80]. Taking the buildings as the basic unit in the residential area, the spatial topology node, the connected state between each building, and a series of variables in the topology diagram are constructed to express the spatial layout characteristics. The systematic clustering algorithm is used to identify the representative residential areas as follows:

Let M and N refer to the numbers of objects and clustering variables, respectively. For any 1≤i≤M, object i is denoted as $X_{i\;}$= ($x_{i1}$, $x_{i2},$ …, $x_{iN}).$ For any two objects $X_{i}$ and $X_{j}$, the square Euclidean distance $d_{ij}$ is computed to measure the degree of the similarity between the i and j objects

(14) $$d_{X_{i}X_{j}}=\sqrt{\sum_{n=1}^{N}{\left(X_{in}-x_{jn}\right)}^{2}}$$

Based on the calculated $d_{X_{i}X_{j}}$, the average distance between two classes of individuals is used as the criterion to determine whether or not to merge the two classes into a new class:

(15) $$D\;(G_{p},G_{q})≜\frac{1}{n_{p}\;n_{q}}\sum_{i\epsilon G_{p}}\sum_{j\epsilon G_{q}}d_{X_{i}X_{j}}$$

Secondly, for each representative residential area, based on the determination of the wind resistance coefficient of the residential area, the source and target points were set based on the wind direction; the minimum-cost path from the source point to the target point was calculated according to the minimum-cost distance model, and a residential area ventilation corridor was constructed [81,82,83,84].

The path cost (D) is used to describe the cost accumulation of a raster path between the start and end points:

(16) $$D=\sum_{i=1}^{n-1}C_{i}\;=\sum_{i=1}^{n-1}f\left(p_{i},p_{i+1}\right)$$

where $p_{i}$ is the vector of the raster pixels, and $C_{i}$ is the neighboring cost between each pair of adjacent raster pixels in the raster path, representing the lowest cumulative cost of any pixel to the nearest and lowest cost destination.

The minimum-cost ($P^{*}$) path is a raster path with the minimum path cost between the starting point $p_{0}$ and ending point $p_{n}$:

(17) $$P^{*}=\arg\underset{P_{j}}{\min}D_{j},\;j=0,\;1,\;2,\;\ldots ,\;m$$

where *m* is the number of grid paths between the start and end points.

The proportion $R_{a}$ of the NVP in residential areas is calculated as follows:

(18) $$R_{a}=\frac{AP}{Area}$$

where *AP* is the total area of the cost path of the residential area, and *Area* is the total area of the residential area.

Because the wind direction determines the source and target points, the NVP of the residential areas is calculated as follows:

(19) $$V_{p}=\sum_{i=1}^{n}W_{f}*W_{i}*R_{a}$$

where $V_{p}$ is the NVP of the residential area, $W_{f}\;$ is the frequency of the wind, $W_{i}\;$ is the intensity of the wind, $R_{a}$ is the proportion of the area of the ventilation corridor in the residential area, and *i* = 1, 2, *n* is the wind direction in the area.

#### 2.3.3. Kernel Density Estimation

Kernel density estimation is a spatial analysis method that estimates the unknown density functions based on the distribution characteristics of the research object in probability theory [85]. By calculating the discrete point of density within a certain window range and using it as the center value of the window, the point is obtained. The spatially-continuous density-change layer of the data strengthens the spatial distribution mode, which can intuitively express the degree of agglomeration of the spatial distribution of residential areas [86,87,88,89]. Compared with the traditional point-density estimation method, kernel density estimation considers the difference in the concentration’s intensity at different positions and higher spatial continuity, and is calculated as follows:

(20) $$f\left(x\right)=\frac{1}{nh}\sum_{i=1}^{n}K\left(\frac{x-x_{i}}{h}\right)$$

where $f\left(x\right)$ is the estimated value of the core density of the residential area, *n* is the sample number of the residential area, h is the search radius, K is the kernel function, and $\left(x-x_{i}\right)$ is the estimated distance between the two residential areas. The higher the value, the greater the density of the spatial distribution of residential areas and vice versa.

## 3. Results

### 3.1. Four Fundamental Residential Area Layouts

**Figure 2:**
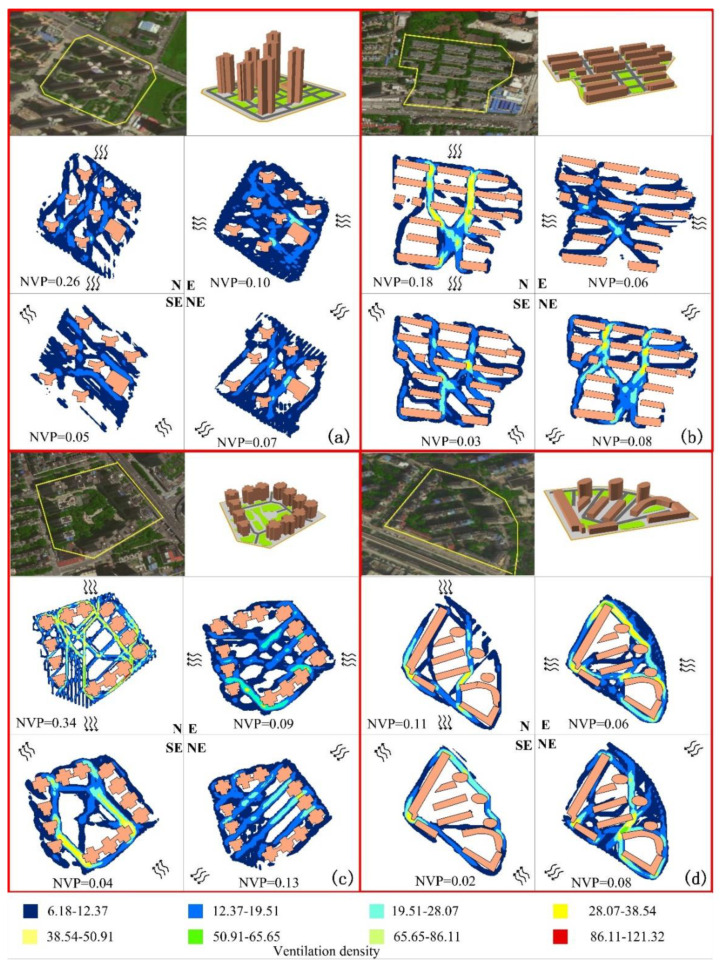
Spatial layout of four representative residential areas: (**a**) points layout, (**b**) parallel layout, (**c**) center-around layout, (**d**) mixed layout; the density of ventilation corridors, and the NVP of each wind downward (N: north wind; E: east wind, SE: southeast wind, NE: northeast wind).

The planning and design of the residential area layouts were determined based on a comprehensive consideration of factors, such as land use conditions, space environment, users, layout methods, and group combinations [90]. After the satellite image recognition, this study selected four representative residential areas in Wuhan, as shown in Table 1 and Figure 2.

**Figure 3:**
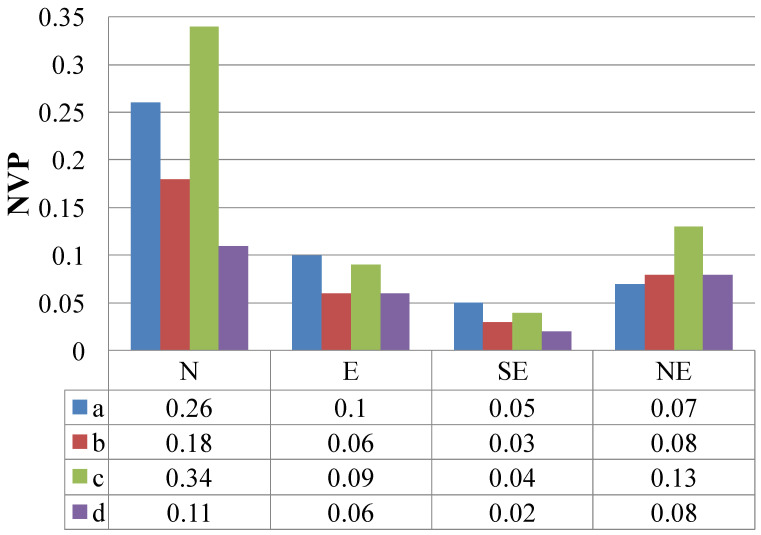
NVP levels at different wind directions for four representative residential areas (a. points layout, b. parallel layout, c. center-around layout, d. mixed layout) (N: North wind; E: East wind, SE: Southeast wind, NE: Northeast wind).

Equation (17) calculates the NVP of each spatial layout with the different downward winds (Figure 2). When the wind direction was northerly, the center-around layout and points layout NVPs were higher at 0.34 and 0.26, respectively, and the NVP of the parallel and mixed were low at 0.18 and 0.11, respectively (Figure 3). When the wind direction was northeast, the center-around layout had the highest NVP, at 0.13; the mixed and parallel had the next highest NVPs, both of which were 0.08, and the point layout was 0.07. When the wind direction was easterly, the NVP of the point and center-around layouts were still in the top two positions, at 0.10 and 0.09, respectively; the parallel and mixed NVPs were lower, with both at 0.06. When the wind direction was southeast, the NVPs of the point layout, center-around layout, parallel layout, and mixed layout were 0.05, 0.04, 0.03, and 0.02, respectively. The total NVPs of the mixed and parallel layouts were 0.27 and 0.35, respectively, and set as NVP I and NVP II, respectively, and the total NVP of the point layout was significantly higher than that of the mixed and the parallel layouts, which were both 0.48, which was set to NVP III. The center-around layout had the highest total NVP of 0.60 and was set to NVP IV.

### 3.2. Assessment of Natural Ventilation Potential of Residential Areas in Wuhan

#### 3.2.1. Spatial Distribution in Districts

**Figure 4:**
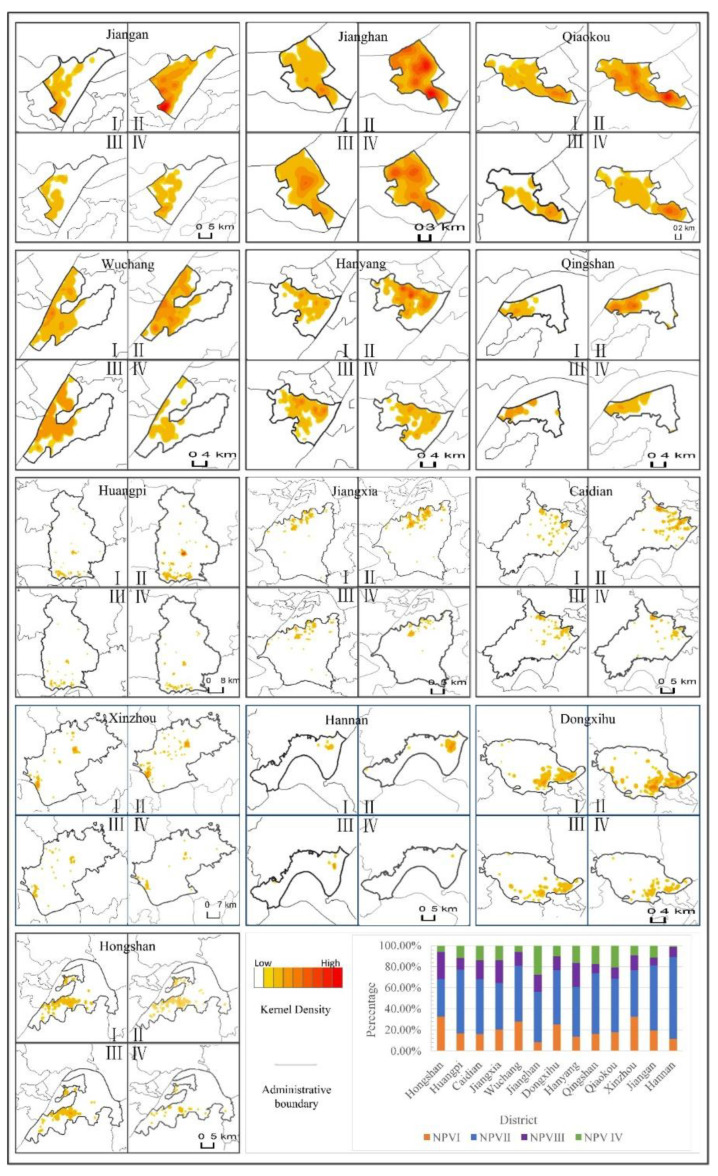
Kernel density and percentage of residential areas with different levels of NVP in each administrative district of Wuhan.

Using the kernel density analysis tool, after several experiments, the space kernel density of the residential areas with different levels of NVP in Wuhan was analyzed with a search radius of 10 m. Furthermore, the equal interval segmentation method was used to divide the kernel density value into nine levels. The purpose of this study was to explore the types of agglomeration and the differences in the distribution of the density gradients of residential areas with different levels of NVP. Therefore, the density ranges from low to high were defined as 1–9 for each level; the kernel density of each level was expressed in the figure according to the gradual color, and it was superimposed and analyzed with the administrative base map. The results of the analysis are presented in Figure 4.

The agglomeration phenomenon of the NVP II residential areas in Jiang’an District was the most significant. There was a high-density center and four secondary high-density centers. The high-density center was located in the southwest of the area, accounting for 61.87% of the total number of residential areas in the area. There was a low-level agglomeration center in the southwest of the NVP I residential areas, accounting for 19.60% of the total number of residential areas in the district. The NVP III and NVP IV residential areas were also mainly distributed in the southwest of the district; the agglomeration phenomenon was not obvious, accounting for 7.20% and 11.33% of the total number of residential areas in the district, respectively.

The agglomeration phenomenon of the NVP II residential areas in Jianghan District was particularly significant, with two high-density agglomeration centers and multiple secondary high-density agglomeration centers. The agglomeration center had a large core density, rich hierarchical levels, and drastic changes, accounting for 47.74% of the total number of residential areas in the district. The agglomeration characteristics of the NVP IV residential areas were also obvious. The two high-density agglomeration centers were connected at the edge and formed a saddle shape, accounting for 27.46% of the total number of residential areas in the district. The NVP III residential area had a relatively obvious agglomeration center located in the center of Jianghan District; however, the grading decline was more moderate, accounting for 16.45% of the total number of residential areas in the district. The NVP I residential areas were agglomerated in the southeastern part of the district, and the core density of the agglomeration center was not high, accounting for 8.34% of the total number of residential areas in the district.

The agglomeration phenomenon of the residential areas with NVP II in Qiaokou District was the most obvious, with a high-density agglomeration center and three low-level agglomeration centers. The densest agglomeration center was located in the southeast of the area, accounting for 51.13% of the total number of residential areas in the area. The NVP IV, NVP I, and NVP III residential areas had an obvious agglomeration area, which was the same location as the NVP II residential areas, accounting for 20.60%, 17.74%, and 10.53% of the total number of residential areas in the district, respectively.

The NVP II residential areas of Wuchang District formed multiple continuous agglomeration centers in the central and western regions of the district, with a wide distribution range, accounting for 52.90% of the total number of residential areas in the district. The NVP III residential areas were concentrated in the western part of the district, forming a large-scale secondary high-density area; the grading change was not obvious, accounting for 13.09% of the total number of residential areas in the area. The NVP I residential area had a secondary high-density center and two low-level agglomeration centers, and the core density of the agglomeration center was not high, accounting for 28.07% of the total number of residential areas in the district. Residential areas with NVP IV were also mainly distributed in the central and western regions of the area, without obvious agglomeration, accounting for 5.94% of the total number of residential areas in the area.

There were two high-density agglomeration centers in the Hanyang District NVP II residential areas located in the northeast of the district. The two high-density agglomeration centers were connected at the edge, accounting for 47.42% of the total number of residential areas in the district. The NVP III residential areas were composed of three secondary high-density agglomeration centers and were distributed in a “2 + 1” pattern, accounting for 22.79% of the total number of residential areas in the district. There were several low-level agglomeration centers in the residential areas of the NVP I and NVP IV residential areas of the ventilation potential. The changes eased, accounting for 13.63% and 16.15% of the total number of residential areas in the district, respectively.

There were two high-level agglomeration centers in the NVP II residential areas of Qingshan District, which were located in the middle and western parts of the district, and were adjacent to each other. This accounted for 57.61% of the total number of residential areas in the district. The NVP III residential areas were concentrated in the northwest of the district, accounting for 8.66% of the total number of residential areas in the district. The NVP I and NVP IV residential areas had a low-level agglomeration center, accounting for 16.42% and 17.31%, respectively, of the total residential areas in the district.

The agglomeration of the residential areas at the four levels of NVP in the above administrative district was notable. In other districts, such as Huangpi District, Jiangxia District, Caidian District, Xinzhou District, and Hannan District, the distribution of the residential areas in the district was relatively scattered, with no obvious agglomeration centers, or they only had local distribution.

#### 3.2.2. Quantity and Change

There were 4203 NVP II residential areas in Wuhan, accounting for 50.46% of the total, which was more than half. The NVP I residential areas were second, at 1676, accounting for 20.12% of the total. A total of 1312 NVP III residential areas accounted for 15.75% of the total. The number of NVP IV residential areas was the least at 1139, accounting for 13.67% of the total. According to the results in Figure 4, regarding the number of residential areas in Wuhan, the order was NVP II > NVP I > NVP III > NVP IV. The residential areas with NVP II and NVP I were the first and second highest percentages, and the sum of the two was 70.58% of the total, which was significantly higher than the residential areas with NVP IV and III. Furthermore, the number of residential areas with the best ventilation was the smallest. The ventilation in Wuhan’s residential areas was not optimal and must be improved urgently.

**Figure 5:**
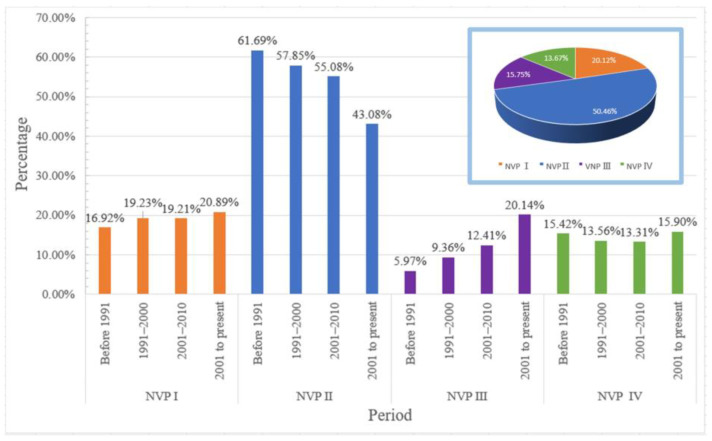
Percentage and change in residential areas with different levels of NVP in Wuhan.

To assess the trend of changes in the ventilation situation of residential areas in Wuhan over time, this study divided the construction years of the residential areas into four time periods: before 1991, 1991–2000, 2001–2010, and 2011. From the statistical results in Figure 5, it can be observed that the percentage of residential areas with NVP I increased from 16.92% before 1991 to 20.89% from 2011 to the present, with a growth value of 3.97%, which is a large increase. The NVP II residential areas accounted for the largest percentage of the four spatial layouts in the four time periods, up to 61.69% before 1991, although there was a decline in the four time periods from 61.69% before 1991 to 43.08% for the interval from 2011 to the present; however, the overall percentage is still large. The percentage of residential areas with NVP III increased during the whole time period. The residential areas with NVP IV accounted for a relatively small percentage of the four spatial layouts in the whole time period at <20%, and showed a downward trend in the first three periods, from 15.42% before 1991 to 13.31% during 2001–2010. It rebounded from 2011 to the present, reaching 15.9%; however, the overall growth rates are not obvious at only 0.48%.

## 4. Discussion

(1) With the improvement in living standards, the environment of residential areas has attracted increasing attention [91,92]. The pandemic will not end in the short term [93]. Wearing masks, washing hands frequently, and maintaining social distancing are the new normal during the COVID-19 pandemic. To reduce the likelihood of transmission via air, the WHO recommends a natural ventilation rate of at least 60 L per second and per person and at least six air changes per hour [94]. Hu Tingting et al. indicated that a higher ventilation efficiency was found in small-building coverage ratios and large passage widths by evaluating the ventilation efficiency through the visit frequency, the space-average normalized concentration, and the space-average wind speed ratio [95]. Through a prediction model between the ventilation efficiency of residential areas and the building form, the largest impact of the height fall morphological index on the wind speed ratio at different heights was found by Feng Wei et al. [96]. The results of this study confirmed that a reasonable spatial layout of residential areas could effectively improve ventilation, thereby enhancing the ability of residential areas to resist the spread of COVID-19.
(2) The spatial distribution of the residential areas in Wuhan has obvious agglomeration characteristics. The study by Zhang Xiaole et al. found that the dilution of the surrounding air can rapidly reduce the risk of aerosol-borne infections [43]. We found that the concentration of residential areas in the central urban area (e.g., Wuchang District) was much greater than that in the surrounding areas. During the pandemic, Wuchang District was the hardest hit area in Wuhan. In addition to the fact that the South China Seafood Market was located in Wuchang District, COVID-19 spread rapidly throughout the country from Wuchang District because the residential areas in Wuchang District were highly agglomerated, and people had frequent contact with each other, which also makes transmission prevention more difficult. Therefore, reasonable planning of the concentration of residential areas is another way to avoid the virus’s rapid spread in the future.
(3) With the acceleration of urbanization, the number of residential areas in Wuhan has been increasing since 1991. However, the NVP of the Wuhan residential areas has not significantly improved. According to the WHO, the “Healthy City Project” is a long-term sustainable development project [97]. Its goal is to include environmental issues on the agenda of urban decision-makers, prompt local governments to formulate corresponding healthy environments, and thereby improve the health of residents. Improving the ventilation of residential areas is one way to achieve a healthy environment. In our study, at present, the overall ventilation status of Wuhan’s residential areas is not optimistic, and there is substantial room for improvement. Relevant experts highlighted that the COVID-19 pandemic would not end in a short time, and the ongoing fight against the pandemic has a long way to go. This requires urban planners to pay attention to the ventilation between houses in future residential area planning, improve the ventilation conditions to improve the ability to resist viruses, such as COVID-19, and provide protection for people’s future healthy lives.
(4) The rational development and utilization of big data will open up a new cognitive space, propose new models for solving various practical problems, give rise to an unprecedented new field of the digital economy, and create a new human lifestyle [98,99]. Urban big data promotes the transformation of urban planning to refinement and quantification [100,101,102] and provides a new perspective on urban cognition and new technologies and methods for urban planning [103,104]. The use of big data, combined with GIS technology to measure the human settlement environments, can provide an in-depth analysis of the internal differences in the human settlement environment and provide a scientific theoretical basis and important auxiliary services for pandemic prevention and control [105,106]. Using big data technology to mine relevant data, such as urban residential areas, can effectively supplement traditional data. Having sufficient research samples is key to ensuring the scientific nature of the research results. Big data, as the main data source in this research, provides information support for the spatial layout analysis of this research. The layout of residential areas is an important factor in the assessment of the NVP of residential areas. Therefore, the role of big data in preventing and controlling the COVID-19 pandemic should not be underestimated.
(5) As a city in southern China, Wuhan, in our study, has obvious regional characteristics, which lead to some representative residential areas. Globally, the residential areas in different climatic zones (such as the tropical, temperate and frigid zones) and countries and regions with different topographic and geological conditions (such as mountains and plains) are quite different. Each country or region will form some representative residential areas with local and regional characteristics under the combined action of these environmental factors [107,108]. For example, in China, the north and the south are shaped by natural conditions, such as the heat and terrain, culture and other human factors, to form different residential areas [109,110,111]. Nevertheless, in this paper, the general framework of POI picking based on the semantic similarity model, representative residential areas of identification, the ventilation LCDM model’s development, and the construction of a ventilation evaluation system could benefit local planning officers and policymakers in the different typologies of districts.

## 5. Conclusions

This research analyzed the spatial layout of urban residential areas based on large geodata and evaluated the NVP of four typical spatial layouts based on the LCDM. The study found that, in the susceptible seasons of winter and spring, when the outbreak of the pandemic occurred in Wuhan, the NVP of the residential areas with the center-around and points spatial layout was better than that of those with mixed and parallel layouts. However, the current residential areas in the study area were still dominated by the parallel spatial layouts with low-ventilation effects, and the proportion of residential areas with higher NVPs in the center-around and point spatial layouts was low. This research suggests that in the design of future residential areas, priority should be given to the center-around and point spatial layouts to improve the overall NVP and the ability to resist airborne viruses.

Under the normalization of the pandemic, urban residents have increasingly higher requirements for their living environment. A higher-ventilated building can help control the spread of diseases such as COVID-19. Our research found that the Jianghan, Qiaokou, Jiang’an, Hanyang, and Wuchang districts, as the central urban areas of Wuhan, comprise most of the residential areas in Wuhan; however, the parallel and mixed residential communities with low NVPs have a wide agglomeration range and strong agglomeration, and the ventilation status is not optimal. This enlightens planners to consider the environmental elements of a central city in urban planning and design, such as the NVP of residential areas, so as to design an environment that is more conducive to healthy living for residents.

In addition, our research found that the overall NVPs of Wuhan’s residential areas did not greatly improve over time. Although the number of center-around residential areas with a good NVP increased over the study period, the growth value was only 0.48%, while the number of mixed residential areas with a low NVP had a growth rate of 3.97%. The delineated residential areas with a low NVP declined overall, but they still accounted for a large percentage. This suggests that strengthening and improving the ventilation of residential areas cannot be accomplished in a short-term duration, and a substantial effort is required.

Our work did not incorporate Wuhan’s COVID-19 cases during the outbreak into the NVP assessment due to inadequate data availability at the residential area level. This is similar to other research. For example, still lacking COVID-19 cases, research by Li and Tang proposed an improved Wells–Riley model to predict the infection risk of COVID-19 in the main functional space of an outpatient building [112]. The results showed that the semi-enclosed hospital street design could improve natural ventilation, further dilute viral aerosols, and reduce the risk of infection to a negligible level (below 0.04%). In the future, COVID-19 case data at the residential area level can be used to prove whether or not there is a link between the spatial features of residential areas, their NVP level (I, II, III, and IV), and the number of cases, so as to enhance the authenticity and practicality of current research.

This study conducted a static pattern evaluation on the NVPs of four representative residential areas in the early stage of the severe epidemic situation in Wuhan. Such a static evaluation is similar to Fan’s and Park’s research. Fan et al. found that having moderate ventilation is an important means of reducing the pollutants’ concentration [113]. Park et al. selected fifteen occupied single-family dwellings from three sites to quantify the effects of natural ventilation systems on particle concentrations in residential areas [114]. However, there are some ongoing issues that require continued work for conducting virus propagation dynamics evaluation, e.g., how does the virus propagation change with the wind direction? What are the differences in virus propagation in residential areas with different layouts? Therefore, in future research, it is necessary to incorporate dynamic virus propagation into the assessment framework to improve the depth of the research.

## Figures and Tables

**Table 1:** Spatial layout characteristics of four different residential areas.

Residential Area Layouts	Points Layout	Parallel Layout	Center-Around Layout	Mixed Layout
Feature	Refers to the layout of single-yard houses, multi-storey point-type and high-rise tower buildings, with public buildings, central green spaces, etc., surrounding them	Panel building units are arranged in rows according to a certain orientation (generally north-south orientation)	The building volume is arranged along the street or courtyard, forming a closed or semi-enclosed inner courtyard space	Combination of three basic forms or combinations of deformations
Representative Residential areas	Guangdian Lanting Shenghui is located in Hanyang District, Wuhan, 114.23° E and 30.52° N. It was completed in 2016 and has a total area of 43,128.08 m^2^.	Lu Zong Garden, located in Wuchang District, Wuhan City, 114.32° E and 30.52° N, was built in 2001, with a total area of 40,315.29 m^2^.	Xinhua Homes, located in Hankou District, Wuhan, 114.26° E and 30.63° N, was built in 2005, with a total area of 61,867.51 m^2^.	Hankou Spring-Xingyuan, located in Qiaokou District, Wuhan, 114.20° E and 30.60° N, with a total area of 35,321.01 m^2^.

## Data Availability

The datasets generated and/or analyzed during the current study are available from the corresponding author on reasonable request.
